# Advancements in Family-Based Treatment of Adolescent Anorexia Nervosa: A Review of Access Barriers and Telehealth Solutions

**DOI:** 10.3390/nu17132160

**Published:** 2025-06-28

**Authors:** Ashlea Hambleton, Daniel Le Grange, Stephen Touyz, Sarah Maguire

**Affiliations:** 1 InsideOut Institute, University of Sydney, Sydney Local Health District, Sydney, NSW 2000, Australia; 2 UCSF Weill Institute for Neurosciences, University of California San Francisco, San Francisco, CA 94107, USA; daniel.legrange@ucsf.edu

**Keywords:** anorexia, family-based treatment, telehealth

## Abstract

Anorexia Nervosa (AN) is a psychiatric illness with serious medical and physiological implications. Anorexia Nervosa is characterised by significant disruptions in weight, growth and physical health resulting from disordered behaviours such as food restriction, purging and inappropriate exercise. The illness is associated with substantial physical, psychological, social and economic burdens affecting all areas of functioning. Typically emerging in adolescence, AN can have a chronic course and high risk of mortality, with evidence suggesting that approximately 10% of individuals diagnosed with AN will die from medical complications or completed suicide. Whilst inpatient treatment reduces mortality risks through nutritional and weight restoration, outpatient treatment is the preferred level of intervention. In the case of adolescents, family-based treatment (FBT) is the recommended and most researched outpatient model for medically stable adolescents. However, access to FBT is limited, and there are several barriers that exist to receiving care from trained clinicians. This review provides a literature update on studies reporting the real-world access challenges for FBT, with particular attention paid to non-research settings. The review also highlights how digitally delivered treatment, specifically telehealth, has been used to increase access to FBT and examines the preliminary outcomes of telehealth-delivered FBT, which appear comparable to traditional in-person care. Despite these promising findings, provider, intervention and systemic factors have challenged the delivery of traditional in-person and telehealth FBT in real-world settings. Critical areas for future research include the need to understand the impact of potential confounders and what adaptions may be required to increase model feasibility in community settings, where access to specialist services is often limited and access challenges are most felt.

## 1. Anorexia Nervosa

Anorexia Nervosa (AN) has garnered a reputation as one of the most dangerous, debilitating, and life-threatening among all psychiatric conditions. Anorexia Nervosa is considered “difficult to treat”, with relatively low rates of treatment engagement, remission and sustained recovery [1] and high rates of mortality as a result of severe medical and psychiatric complications [2]. Consequently, improving the detection of AN, understanding the barriers to accessing care, and improving the outcomes of adapting evidence-based treatments that aim to improve reach have been a core focus of clinical research.

Diagnostically, AN is characterised by a significant disruption to normal eating behaviours that interferes with weight growth or maintenance, alongside a fear of weight gain and a disturbed perception of the weight and/or shape of one’s own body [3]. The Diagnostic and Statistical Manual for Mental Disorders, 5th edition, text revision (DSM-5-TR) describes two subtypes of AN: the restricting type (AN-R), where individuals primarily achieve weight loss or control through dieting, fasting and/or excessive exercise, and the binge–eating–purging type (AN-BP), where in addition to food restriction and weight loss and control behaviours, individuals also engage in binge eating and recurrent episodes of self-induced vomiting and/or the misuse of laxatives, diuretics or enemas. Some individuals present with symptoms consistent with an AN diagnosis (e.g., food restriction, significant weight loss, an intense fear of weight gain, and distorted perceptions of the body) while their weight remains within or above the normal range.

### 1.1. Medical Consequences of Anorexia Nervosa

While formally classified as a psychiatric illness, the physiological and medical consequences of AN can be chronic and life-threatening. Food restriction and malnutrition are associated with numerous medical complications and comorbidities that impact all body systems [2,4]. Cardiac complications, including arrhythmia, bradycardia, heart failure and hypotension, are the primary cause of death in AN [5]. Additionally, individuals are faced with an increased risk of endocrine complications (functional hypothalamic amenorrhea females and low testosterone in males), neurological problems, digestive complications and disorders, dehydration and electrolyte abnormalities [6,7]. Other complications include decreased muscle mass, a loss of energy, disrupted sleep, difficulties maintaining an adequate body temperature, lanugo, brittle hair and nails and hair loss [6,7].

### 1.2. Psycho-Social Symptoms and Comorbidities of Anorexia Nervosa

Individuals with AN display premorbid cognitive difficulties, including abstract, obsessive and rigid thinking, attention and concentration [8]. During illness, these cognitive traits are believed to play a key role in developing and maintaining preoccupations with food, eating, and the body, which can lead to body checking (monitoring, scrutinising and comparing one’s body weight or shape) and body avoidance behaviours. These behaviours reinforce body image distortions, with visual and metric body size estimation tasks demonstrating that individuals with AN perceive their bodies to be bigger than they really are [9]. Low self-esteem, self-worth, perfectionism and high levels of self-criticism are risk factors for the development of AN [10] and remain markedly pronounced during illness. Individuals with AN have been found to have lower reflective functioning levels (the ability to discern between external and internal reality, emotional and intrapersonal processes and perceived mental states are subjective and changeable) and difficulties recognising the emotions of others [11]. Further, these deficits appear to also be present in the parents of adolescents with AN [12].

Rarely does AN present in isolation, with between 50% and 97% of people diagnosed with AN experiencing at least another psychiatric disorder [2]. A large population-based study (N = 3,186,133) identified a bidirectional relationship of comorbidity where the risk of AN was elevated among those with prior psychiatric diagnoses and vice versa [13]. The elevated risk of other psychiatric conditions appears to continue after AN recovery [1]. Anorexia nervosa most frequently co-occurs with affective/mood disorders (comorbidity rates up to 67%; [14]) and anxiety disorders (comorbidity rates up to 64%; [15]), with obsessive–compulsive disorder and social phobia being the most common subtypes [16]. Childhood trauma, personality disorders, substance use disorders and schizophrenia are also relatively common co-occurring conditions known to influence the expression of AN symptomatology, the course of illness and treatment outcomes [17]. Psychiatric risk is heightened by the elevated mortality of AN, with individuals diagnosed with AN 32 times more likely to die by suicide compared to the general population [18], amplifying the crucial need for effective and timely treatment.

## 2. Family-Based Treatment of Anorexia Nervosa in Young People

Given the severe risks of AN, there is an imperative to deliver treatment as quickly as possible after the onset of illness [19]. International evidence-based clinical practice guidelines recommend four priority treatment areas for young people with AN; (1) medical—adequate weight gain and return to physical health; (2) nutritional—the resumption of normal eating and meeting nutritional needs for development; (3) psychological—the resolution of distorted cognitions, body image, self and comorbid issues; and (4) family—addressing barriers to developmentally appropriate individuation, family dynamics and conflict issues [20,21,22]. Family-based treatment (FBT; [23]) is the most researched intervention for adolescents not facing acute medical instability and can be delivered within inpatient, outpatient, day programs and in-home treatment settings.

### 2.1. Overview of Family-Based Treatment

The publication of the treatment manual Family-Based Treatment (FBT) for Anorexia Nervosa [23] was another significant step forward in the treatment of adolescent AN. Born out of the work conducted at the Maudsley Hospital in London, FBT similarly emphasises unified parental support and intervention as the primary resource to facilitate the recovery of adolescents with AN. Family-Based Treatment comprises three treatment phases with distinct goals and tasks. The transition between phases is fairly fluid and clinically determined [23]. In phase one, the young person’s parents are charged with the responsibility of managing eating disorder behaviours and establishing a regular pattern of eating to facilitate weight gain within the home environment. In phase two, parents work collaboratively with the therapist, negotiating the gradual and age-appropriate transition of the responsibility of eating back to the young person. In phase three, relapse prevention and normal adolescent developmental issues become the focus of treatment. Phase three might also involve referral to individual or general family therapy post-FBT to address comorbid or systemic issues.

To deliver FBT, clinicians first complete specialised training, usually through a multi-day workshop and supervision. Given the psychiatric and medical risks of AN, treatment is most safely delivered within the context and support of a multidisciplinary team [24]. Prompt refeeding is prioritised to avoid the medical consequences of malnutrition and a low body weight [4]. Initially, FBT appointments are scheduled weekly and the frequency generally reduces as the adolescent becomes progressively well in later phases [23].

### 2.2. Effectiveness of FBT

Family-Based Treatment is the most well-researched treatment for adolescents with AN [25,26]. Meta-analytic reviews of RCTs have systematically demonstrated the effectiveness compared to control groups in restoring adolescent weight and reducing ED symptoms [27,28,29,30,31,32,33,34,35]. A comparison of the efficacy of FBT with individual therapy found no difference between models at the end of treatment [25]. However, FBT emerged as the superior treatment when including follow-up data [25]. Only one of the three studies in the meta-analysis evaluated FBT for AN (the other two examined FBT for Bulimia Nervosa [BN]). Conversely, a subsequent meta-analysis of studies conducted until mid-2018 noted a small amount of low-quality evidence supporting the effectiveness of family therapy approaches to achieve remission at the end of treatment compared to individual therapy models. This difference was maintained at 12-month follow-up [36]. The review noted an overall lack of evidence demonstrating a clear advantage of family therapy compared to individual or education interventions.

A recently published systematic review and meta-analysis of the updated evidence base (inception—January 2024) examined the outcomes of FBT, FT-AN and behavioural family therapy (BFT; [37]). The authors reported that the weight outcomes at the end of FBT were superior to individual therapy, but this effect declined below statistical significance at follow-up. The authors speculate that their contrasting results to Couturier and colleagues [25] are reflective of methodological differences, such as their definition of follow-up (from the start of treatment) compared to Couturier et al. [25], who defined follow-up as the time after the end of treatment. Austin and colleagues’ [37] meta-analysis compared four studies reporting the outcomes of family treatments and individual therapy (AFT = 3 and CBT = 1). The analysis found that family treatments were superior to individual therapy at the end of treatment for changes in weight, but not remission or eating disorder psychopathology (although family treatment was favoured). The superiority difference diminished over time, with a non-significant difference at long-term follow-up, again favouring family treatment. Three trials compared conjoint family therapy with separated family therapy, and weight and remission outcomes were statistically superior to those of the separated approach [37].

Modifications, adaptions and adjuncts to FBT have been tested to expand the applicability and improve the recovery rates and outcomes associated with this treatment [38]. Modifications include delivering FBT within higher levels of care such as inpatient and partial hospitalisation programs [39]; adding modules from other evidence-based treatments such as CBT [40], DBT [41] or art therapy [42]; delivering FBT digitally via telehealth or guided self-help [32]; modifying the dosage of FBT [43]; and changing which family members attend FBT sessions with a separated, parent-only form [30]. For example, an RCT compared the relative efficacy of a parent-only form of FBT (PFT) to FBT and found that remission rates were significantly higher in PFT than FBT [30]. However, this difference disappeared at the 12-month follow-up, suggesting that both treatments result in significant and clinically meaningful weight gain over the long term. A later RCT compared the efficacy of an adapted FBT called Intensive Parental Coaching (FBT-IPC) to standard FBT and noted no notable differences between groups from baseline to the end of treatment, with both groups increasing in weight [34]. A scoping review of 43 studies (N = 10 focused on parent only treatments) concluded that weight gain occurred faster with parent support interventions. Parents reported increased self-efficacy in these interventions, which may have implications for parental mental health [38].

The remission rates for FBT somewhat depend on the definition of remission used [44]. When using stringent criteria, defined as achieving ≥95% the expected body weight (EBW) and global EDE score within one standard deviation of community norms [45], the remission rates at the end of FBT range from 22 to 49% [46]. Remission rates increase to 75% when considering clinical and symptom improvement separately, such as only weight restoration (≥95%EBW) or only EDE score [25]. Follow-up studies have demonstrated that these remission rates are maintained up to five years post-treatment [47,48,49,50,51].

An early treatment response defined by weight gain is consistently demonstrated as an important predictor of outcome. Specifically, a weight gain of approximately 2–2.5 kg by week four of FBT is predictive of remission and improved psychological outcomes at the end of treatment [26,31,52]. A recent review of predictors, moderators and mediators of outcome noted the impact of illness severity at the beginning of treatment [26]. Individuals with a longer duration of illness, who were older, more weight suppressed or had experienced previous hospitalisations and ED treatment were associated with poorer outcomes, such as not achieving remission or weight restoration at the end of treatment [26].

Research indicates that FBT is less effective and a longer duration of treatment is required for adolescents with concurrent obsessive–compulsive traits [27,43]. Likewise, separated, single-parent and reconstituted families tend to need more sessions of FBT to achieve remission rates similar to their intact counterparts [43]. Finally, patients with parents that express a high level of emotion and criticism (towards each other and/or the affected child) do better in a parent-focused form of FBT (where the young person is not as involved or present) compared to traditional FBT, which risks the child being exposed to parental criticism in the therapeutic setting [30].

### 2.3. Dissemination and Implementation of Family-Based Treatment

General psychotherapy research suggests that outcomes are usually better when treatment is delivered in research settings [53], with the effect sizes markedly declining once interventions are disseminated and implemented in usual clinical care [54]. While few people affected by AN receive a diagnosis and an even smaller proportion will access treatment [55], those that do generally engage with community-based services rather than research settings [56,57]. So far, meta-analytic data supporting the efficacy of FBT has been obtained from studies conducted in academic and specialised treatment settings where samples were recruited (not clinically referred) and therapists were research employees, not everyday practising clinicians [26,37,58].

Dissemination and implementation FBT research seeks to answer two questions: (1) what are the outcomes of FBT delivered outside of research settings and (2) what are the outcomes of FBT delivered outside of specialist settings and in the community? Relating to the former, two studies in a specialist service compared the outcomes of FBT delivered under research conditions to that in usual clinical care [59,60]. In one study, while both groups achieved positive results, weight restoration took longer for adolescents with more severe AN in non-research conditions [59]. The authors attributed the difference in outcomes to increased therapist and family motivation in the research group that had a reduced wait time between referral and FBT, increased treatment observation through contact with research staff, fidelity monitoring, and focus on achieving good outcomes within a fixed number of sessions [59]. However, a later Australian study utilising a similar design found no difference in outcomes between research and clinical groups accessing FBT from an outpatient ED program [60]. The sites involved in these two studies were clinical services linked with research centres specialising in treating AN with financial and clinical resources that were likely higher than in community treatment settings [61].

The last decade of FBT research has seen an increasing number of studies demonstrating promising remission rates for FBT implemented in real-life clinical settings [61,62]. A study of an Australian tertiary setting noted that AN-related inpatient admissions, readmissions and total bed days were reduced after implementing FBT into their service [63]. The success of the implementation was further evidenced by the sustainment of the model beyond the study period, attributed to an ongoing training, education and supervision program [63]. More recently, a similar study design was used to demonstrate the translatability and successful implementation of FBT in an Asian tertiary paediatric hospital setting [47]. Over two years, young people achieved weight restoration faster than the previous treatment approach (medical monitoring and non-specific therapy). The study also highlighted the challenges of delivering FBT in a clinical setting with real-world confounders such as limited FBT-trained therapists throughout implementation, a lengthy wait time between referral and treatment, and culturally linked stigma.

## 3. Barriers to Accessing Family-Based Treatment

Despite the evidence supporting the use of FBT to achieve significant improvements in core AN pathology, the dissemination and implementation of FBT has been particularly impacted by the research–practice gap (i.e., delays or difficulties disseminating research trial results to clinical practice). Further, families attempting to access FBT in community settings are challenged by the treatment gap (i.e., the discrepancy between those who need FBT and those who can access FBT [64]). Both gaps are exacerbated by factors relating to the intervention (such as intensity and demand on clinicians and families), treatment providers (such as confidence and fidelity), the family and parent (such as parental mental health), systemic factors (such as culture and economy) and geographical barriers.

### 3.1. Intervention Factors

Family-Based Treatment requires an intensive whole-family focus on the young person’s recovery, including the attendance of all family members at every session [65]. To optimise the success of refeeding, FBT therapists recommend that parents take vocational leave and be home full-time for at least the first two weeks of treatment to support every meal their child requires [23]. Qualitative interviews with parents who participated in FBT have described significant overwhelm, anxiety and eventually carer burnout relating to the responsibility of refeeding [66]. Phase 1 can be particularly challenging for families, with heightened family conflict surrounding meals, with “plates smashed, tables turned four hours later and we’d both be exhausted; no food would’ve been eaten” [66]. Further, parents have described frustration with the initial focus on eating symptoms: “When’s the psychology part come in?” [67]. These factors, compounded by an already overwhelmed family system disrupted by AN can contribute towards family reticence to commence FBT [67].

Therapists have also described feeling anxious to deliver FBT due to concerns about how to manage family and adolescent distress [68]. Additionally, therapists have reported it can be tiresome to maintain the level of effort required on their part to build and sustain parental anxiety and commitment throughout treatment, especially as the adolescent gains weight and the urgency may subside [69]. Families may request alternative treatments where parents are less involved, such as individual or inpatient therapy due to fatigue from the initial phase of FBT, a lack of confidence in the treatment model, or a misunderstanding of AN [69].

Therapists have described difficulties treating adolescents presenting with other untreated comorbidities that may maintain or interact with AN within an FBT framework [68]. Additionally, therapists have disagreed with the FBT principle that weight gain should be the initial primary focus of treatment when other concerns such as risk, language barriers and immigration concerns demand prioritisation [70]. For practitioners working in community settings, juggling clinical priorities risks lower levels of FBT fidelity [70].

### 3.2. Provider Factors

Treatment fidelity in FBT, as measured by treatment adherence to the core components of the intervention and the therapist’s competence or skill in delivering the treatment, is typically assessed in studies conducted in academic settings. To date, only one study has found that the level of adherence did not significantly influence adolescent weight changes [71]. Across the sites, the therapists’ average years working with AN (range M = 3 years [SD = 3.4]–M = 11.7 years [SD = 4.2]) and family therapy (range M = 3.5 years [SD = 1.5]–M = 13.2 years [SD = 4.8]) suggested a moderately to highly experienced group. The proficiency of therapist competence was not recorded and could have impacted the non-significant result. However, this was unlikely, given the therapists’ experience levels, completion of intensive training and two pilot cases, attendance of weekly supervision from a site supervisor and monthly supervision with study investigators. These findings mirror the results observed across psychotherapy research. So far, fidelity has had little explanatory impact on treatment outcomes, and the range of fidelity scores has been limited by trial therapists trained and supervised in a way that enhances adherence [72].

An online survey of community-based therapists (N = 117) identified that over one-third have strayed significantly from the FBT treatment manual and evidence-based recommendations [73]. A qualitative study of community-based FBT therapists (N = 9) found the variable utilisation of FBT components; 88.89% of therapists reported to weigh the adolescent each session, 100% externalised AN, 55.56% conducted the family meal and 100% reported to include other therapy models in their treatment approach [74]. Another study of community-based therapists (N = 129) noted that for novice and advanced therapists, attending further training and having a positive attitude towards evidence-based practice predicted intent to practice consistent FBT strategies. Despite this improved intent, the analysis revealed that many therapists continued to use techniques inconsistent with FBT, even after attending specific training [75].

Studies have found that adherence is highest in the early phases of treatment when the FBT model is most prescriptive and behaviourally focused [71,76]. Adherence levels were lower on process-based FBT interventions, such as building sibling or parental alignment and later treatment phases [76]. A recent multisite implementation study examined the treatment adherence of 26 therapists and noted that fidelity was most consistent across sites in Phase 1, with the variability in fidelity increasing in Phases 2 and 3 [71]. The authors hypothesised that this adherence drift in the later phases of FBT was attributable to the goals of Phase 3 being broad and encouraging the exploration of adolescent issues relevant to that individual patient and their family [71].

Previous research has suggested that therapist drift from manualised treatment can be attributed to the therapist’s personal beliefs about FBT or low confidence in delivering the process elements of the model [77]. A qualitative study exploring community-based therapists’ uptake of FBT found that 37% of therapists did not weigh the patient before sessions and only 25% reported regularly completing the family meal session [68]. In another study, therapists reported omitting these components due to a lack of training or confidence and a fear of how contentious the family meal session could become if the adolescent refused to eat [69]. Relatedly, therapists with higher self-reported anxiety are more likely to avoid weighing the patient [68,73].

Whilst the data collected so far suggests that no significant relationship exists between FBT fidelity and outcomes, it is less clear what impact major drift and model omissions might have. Further, no research has examined the treatment fidelity of FBT delivered by therapists without extensive training or ongoing supervision under non-research conditions (i.e., the ‘real-world’). Additionally, non-specialist and community-based therapists are treating AN in conjunction with comorbid psychiatric concerns, which may require greater treatment flexibility and greater ‘drift’ [62,73].

### 3.3. Family Functioning and Psychosocial Barriers

The parents and carers of a child with AN are at an increased risk of depressive and anxious symptoms, isolation from key support networks and poor family functioning [78,79]. These factors have been described by therapists as a contraindication for FBT: “parents are not well enough, there’s some pathology going on with parents there’s just a lot of dysregulation and it’s just not gonna work” [69]. While no study has explicitly demonstrated an association between parental ill mental health and poor FBT outcomes, poorer general family functioning has been predictive of increased comorbid psychiatric conditions, as well as more severe AN symptoms in the affected adolescent [80]. Previous research has linked increased comorbidity and AN severity to poorer FBT outcomes [81,82], highlighting an area for future research to evaluate this potential contraindication.

### 3.4. Systemic Factors

#### 3.4.1. Culture, Values and Diversity

Given the high prevalence of AN among adolescents among minority groups, researchers have turned their attention to the application and adaption of FBT to better suit diverse families [83,84]. A recent study explored the adaptions made by clinicians implementing FBT within publicly funded Medicaid services in the USA [83]. With regard to cultural considerations, clinicians have noted how the religious traditions (such as Ramadan), foods and languages of multicultural families required consideration [83]) and additional time allocated to Phase 1 [84]. Relatedly, clinicians have noted that socio-politics, minority stress and racially based trauma have implications for the delivery of FBT, with flexibility around the session agenda required: “there’s no way with Black families that when they come into my office, we aren’t spending time talking in our session about the police or anxiety or fears around leaving the house” [84].

Little research has examined the application of FBT in non-Western cultures. In Japan, professional society guidelines recommend that adolescent AN treatment be similar to the adult model with little parental involvement [85]. The treatment of AN in Japanese health systems generally involves referral to medical specialists and weight restoration in inpatient settings due to limited specialised outpatient services [86]. Implementing FBT in Japanese outpatient settings has been challenged by several cultural and systematic barriers, such as treatment being previously delivered by medical professionals and traditional gender roles making it difficult to engage fathers in treatment [86]. A commentary paper described two FBT adaptations in response to these barriers: (1) delivering FBT in inpatient settings and (2) shifting the distribution of parental tasks to accommodate families with traditional gender roles [86]. These changes were found to increase implementation success, with the uptake of FBT in the Japanese health system and improved adolescent AN outcomes [86]. Relatedly, the authors of an implementation study conducted in Singapore wondered whether their dropout rate was explained by family values and the culture being incompatible with the demands and principles of FBT [47].

#### 3.4.2. Treatment Costs

For families, the travel and treatment costs, not including the loss of work forgone to attend appointments, can be substantial [70,74,87]. Respondents to an Australian survey were estimated to spend approximately AUD 7500–15,000 AUD year on healthcare (not only FBT), even after accounting for government rebates [88]. However, little data relating to the actual out-of-pocket expenses incurred for individuals and their families to access (such as travel costs, medical appointments) and receive FBT within different health systems and across models of care is available (such as inpatient or outpatient, privately or publicly funded). Certainly, this is an important area for future research.

Socioeconomic inequities are further amplified by evidence of rising prevalence rates of AN in adolescents from lower socio-economic backgrounds [89]. Further, a qualitative study exploring provider-perceived barriers to implementing FBT highlighted the financial stress associated with obtaining the amount of food necessary for refeeding. For example, therapists reported that families getting meals from food banks were hesitant to provide their child with AN a big meal for fear it would be thrown away [70].

### 3.5. Geographical Barriers

Location influences a family’s ability to access healthcare [56]. Evidence suggests that families living in rural areas face additional mental and physical health disparities compared to their metropolitan counterparts. Rural children and adolescents have heightened mortality rates [90], an elevated risk of eating disorders and poorer access to evidence-based care [91]. Health professionals and treatment services are generally concentrated in highly populated urban cities. For example, 90% of Australian psychiatrists have their main practice in a metropolitan area, which can lead to care ratios of 6000:1 in rural settings [90].

The inequities between urban and rural populations become even more striking when considering access to specialist eating disorder services and treatments like FBT [92]. Rural community services are challenged by lower clinician confidence [93], a paucity of skilled and trained providers [94], a higher turnover of healthcare workers [95,96], and higher treatment costs due to the lengthy duration of FBT and its multidisciplinary nature [91]. In rural communities, where specialty care is lacking, generalist primary care providers might be the only health professional adolescents with AN have contact with. Yet, they are often not adequately trained to screen and diagnose AN [97] and evidence suggests that providers will avoid screening when there is a lack of referral pathways [98]. Further, evidence suggests that even with the implementation of micro-skill training, over 60% of clinicians working in a regional service made an incorrect diagnosis, particularly for false-negative AN diagnosis [93].

The absence of locally based FBT therapists in rural areas obliges families to travel long distances to access treatment providers; given the multidisciplinary nature of FBT, each provider might be in a different location, meaning several lengthy trips each week. In an Australian report, 60% of respondents from rural areas said they had travelled to a capital city to receive appropriate eating disorder treatment, noting the following: “places where treatment is available are hours away from where I live and will take up a whole day to drive there, have an appointment, and drive back again. Very exhausting.” [99]. Another respondent described the compromise between proximity to support systems and treatment accessibility: “I was born & grew up in a rural area [and] had to travel to Sydney every week for therapy [and]/or inpatient treatment. I moved to Sydney to have better access to outpatient treatment, but now don’t have family support & the outpatient facility have had to decrease their [hours].”

Evidence suggests that when rural young people do eventually engage with a clinical service, they are more unwell with a poorer physical health status, a lower BMI and higher medical risks and complications compared to their metropolitan counterparts [92]. Consequentially, evidence suggests rural young people require higher levels of care to respond to their poor physical status, with 25% requiring admissions and over two-thirds requiring readmissions to eating disorder wards [94,100]. In another study, 35% of participants had an eating disorder for over three years before entering a treatment service and almost 90% had psychiatric comorbidities [93]. As the outcomes of FBT are most optimistic for young people with a short duration of illness, without previous hospital admissions and with fewer comorbidities, these studies highlight how rural young people may be at an increased risk of a poor treatment response before even accessing a service and the need for future research to identify and test innovative approaches that improve treatment accessibility [91].

## 4. Digitally Delivered Treatment for Adolescents with Anorexia Nervosa

Digital mental health interventions are a promising solution to some of the challenges associated with traditional in-person therapy [101]. Information and communication technologies such as computers, smartphones, and tablets can be leveraged to deliver evidence-based psychological interventions [102]. Digital interventions can be broadly categorised into clinician-led treatments delivered through digital means, such as telehealth, and internet-based self-help programs. This review specifically focuses on the former, emphasising how telehealth can enhance the delivery and accessibility of FBT for families living in rural areas.

Originally developed to provide care to rural and underserved patients [103], telehealth has since been applied broadly as an effective, efficient and economical method for health care delivery [104,105]. Prior to the COVID-19 pandemic, most research examining telehealth use in eating disorder populations had occurred with adults diagnosed with BN and binge eating disorder [106]. An effectiveness study comparing telehealth-delivered and in-person CBT for adolescents with BN found similar remission rates at the end of treatment [107]. Although symptom abstinence was somewhat improved for the in-person treatment group, these results were of marginal clinical significance [107]. It has been hypothesised that the additional medical management and lower treatment engagement rates may be why researchers were hesitant to examine telehealth treatment for AN [108].

### Outcomes of Telehealth-Delivered FBT

Before the COVID-19 pandemic, only two studies with very small sample sizes (Anderson, Byrne [109] N = 10; Goldfield and Boachie [110] N = 1) had examined the outcomes of telehealth-delivered FBT. A pilot study was the first to demonstrate that FBT delivered via videoconferencing was feasible, acceptable, and yielded positive treatment outcomes [109]. The study recruited a small sample size (N = 10) of adolescents diagnosed with AN or AAN living in rural Illinois, USA. Family-Based Treatment was delivered by one highly trained therapist working within a specialist eating disorder service affiliated with a research centre. While the study did not include a comparison group, the outcomes of adolescents at the end of telehealth FBT were fairly equivalent to those reported in in-person studies, with a significant improvement in %mBMI from baseline to the end of treatment (*p* = 0.013) and from baseline to six-months follow-up (*p* = 0.032). There were significant improvements in eating disorder symptoms at both timepoints. The authors concluded that there was no compromise to the effectiveness of FBT by changing the mode of delivery to telehealth and that treatment issues could be managed in the same way they would in vivo [109].

Over 40% of participants enrolled in a recent RCT comparing FBT to FBT with intensive parent coaching (FBT-IPC) for early non-responders (defined as not gaining 2.4 kg by session four) received treatment virtually due to the pandemic [34]. There was no difference between treatment groups at the end of treatment or at the six- and twelve-month follow-up, but overall, participants had achieved significant weight restoration by the end of treatment. While not explicitly measured, the transition to telehealth for some participants did not appear to impact overall outcomes, with remission rates comparable to previous studies [34]. Another feasibility RCT conducted during the first year of the pandemic compared the outcomes of guided self-help FBT (GSH-FBT) and FBT delivered by videoconferencing (FBT-V; [32]). In this study, adolescents were more medically unwell pre-treatment than is typically reported in other studies, with just under 60% of adolescents requiring hospitalisation prior to the study. The attrition rate for both treatments (12.5%) was comparable to previous in-person RCTs [33]. Clinically, participants in both forms of FBT improved in terms of weight gain, eating-related cognitions and anxiety and depression symptoms, with a non-significant number favouring FBT-V in terms of weight and full remission at the end of treatment.

A recent implementation study examined the preliminary effectiveness of the first four sessions of FBT-V in paediatric eating disorder services [111]. While all young people gained weight after four sessions of FBT-V, only one patient reached the effectiveness benchmark (a weight gain of two kilograms) within four sessions. The absence of a comparator and the small sample size (*n* = 5) limit the ability to determine the potential mediating effect of telehealth on the outcome. Finally, an Australian multi-site study explored the outcomes of telehealth FBT implemented in a public rural health system [112]. The study evaluated the success of implementation via the RE-AIM framework, focusing on reach, efficacy, adoption, implementation and maintenance. Approximately two-thirds of eligible families within the health system consented to participate, indicating substantial interest in telehealth FBT and treatment engagement and completion rates above 60%. Over 68% of those who completed treatment (*n* = 20) achieved weight restoration and, among those, 36.8% of met weight and psychological remission. Weight gains were sustained at the six-month follow-up. The study collected fidelity data and noted that community clinicians’ self-reported fidelity ratings were comparable to clinical trials.

These studies demonstrate that telehealth can be a viable, cost-effective [113] and effective delivery mode for FBT. Given the absence of follow-up data, further research is required to understand the longer-term outcomes of telehealth FBT, as well as if and how telehealth is sustained by health services in the post-COVID-19 era. Furthermore, it is unclear who benefits from telehealth FBT and who does not. A treatment preference study design would help discern the relationship between participant and family characteristics, such as comorbidities and parental mental health, as well as barriers like rurality and facilitators like clinician upskilling and organisational support, and their impact on the outcomes of telehealth FBT.

## 5. Clinician and Family Experience of Telehealth-Delivered FBT

Although there is an emerging body of evidence supporting the clinical effectiveness of telehealth-delivered FBT, understanding the experience of telehealth FBT is particularly pertinent given that therapists’ anxiety about managing the potentially distressing aspects of FBT, variable fidelity and parental mental health and attitudes towards FBT have been identified as barriers to accessing FBT in person. One of the first studies to examine the experience of telehealth family treatment from the perspective of young people, parents and clinicians found that telehealth treatment was mostly experienced positively [114]. Young people and parents attending a specialist eating disorder service reported that technology had little impact on the treatment experience or the therapeutic relationship. However, when asked about their preference for online or in-person treatment once COVID-19 restrictions had eased, parents preferred online appointments compared to young people [114]. In the context of the pandemic, families and clinicians described an appreciation for the continuity of care that virtual sessions afforded, while also recognising that there was an element ‘lost’ in the online mode, with “missing nuances and process issues” (clinician), and that telehealth “has made it harder to connect”(young person). Clinicians also described the unique therapeutic benefits when connecting to the home setting: “seeing the families in their home settings provides significant information” [114].

This sentiment was also reported by clinicians in another study, where witnessing family dynamics in the home environment allowed them to obtain a better understanding of the family’s challenges: “when you’re being invited into somebody’s home [during a FBT-V session], there’s a lot of really strong positives if we’re comparing how to do it in the office versus how to do it online, there’s a lot of benefits to be able to do a family meal, for example, and watch the family as they interact in real life in their family home” [115]. Of note, the aspects of telehealth treatment described by therapists as helpful were reported by parents as challenging. Parents were required to take over tasks that would have otherwise been completed in person, such as weighing the young person [115]. As noted in Couturier and Pellegrini [111], parents cannot always obtain this data due to the young person refusing to be weighed. While participants unanimously described the accessibility advantages of telehealth, including reducing time and travel costs, some families found that technology issues impacted the therapeutic experience with disjointed sessions or poor video and audio quality [115].

A recent study explored how young people, parents and clinicians’ perspectives of online treatment had changed in the post-COVID-19 era now that in-person treatment was available again [116]. Similar to the original study by Stewart and Konstantellou [114], young people and parents rated the experience of online treatment positively, with most participants endorsing the feeling that they were understood and that important issues were addressed in online sessions. Online or hybrid treatment became more commonplace post-COVID-19, with just under 80% of parents reporting their sessions were in a hybrid format [116]. Contrary to expectations, this study revealed that young people preferred in-person sessions at the beginning and end of therapy. These studies highlighted the acceptability of telehealth-delivered FBT for families, adolescents and clinicians. Further research is required to understand the acceptability and experience of telehealth FBT outside of specialist settings where treatment expectations and needs might be more complex.

## 6. Future Directions for Family-Based Treatment of Adolescent Anorexia Nervosa

While FBT remains the most well-supported outpatient treatment for adolescents with AN, several important questions remain regarding how this model can be optimally adapted, scaled, digitalised, and sustained beyond specialist settings. A small but growing number of studies have demonstrated that telehealth-delivered FBT is feasible and associated with positive clinical outcomes. However, further research is needed to better understand the potential and limitations of telehealth FBT, particularly in community-based services where most adolescents with AN seek care. While early findings suggest no significant compromise on effectiveness when FBT is delivered virtually, most studies to date have been limited by small sample sizes, an absence of comparison groups, and delivery by highly trained clinicians operating within well-resourced or specialist contexts. It remains unclear whether similar outcomes can be achieved when telehealth FBT is implemented by generalist clinicians working in resource-limited, community-based settings.

Future research should also focus on identifying which families are most likely to benefit from FBT-V and which may require alternative or augmented forms of care. Treatment moderators such as comorbid mental health issues, the duration of illness, socioeconomic stressors, and parental mental health remain largely unexplored in the context of virtual delivery. Additionally, research is needed to investigate the role of treatment preferences, digital access and fluency, and cultural values in shaping engagement and outcomes. These insights would help inform a more personalised and equitable approach to FBT delivery.

There is also a need for implementation research to explore how telehealth FBT can be sustainably adopted and delivered in real-world practice. Studies should examine training models, fidelity monitoring approaches, workforce requirements, and cost-effectiveness over time. In particular, understanding how fidelity is maintained, or compromised, by therapists with varying levels of FBT training working without specialist supervision is an urgent priority.

## 7. Conclusions

Anorexia Nervosa is a severe illness with substantial psychiatric and physical impacts and high rates of morbidity and mortality [1]. Young people are particularly vulnerable to AN, with illness onset most often occurring during the adolescent development period due to biological, socio-cultural and psychological risk factors [117,118]. Given the poor outcomes of untreated AN, there is an imperative to deliver effective and safe treatments to adolescents as quickly as possible [1]. Fortunately, FBT has developed an evidence base as an efficacious treatment for adolescent AN [37], with outcomes especially positive for adolescents with a short duration of illness (less than three years) and fewer comorbidities [26].

The dissemination of FBT beyond research settings has been limited by the research-practice and treatment gaps [119], with intervention, provider, family, systemic, geographic, and illness factors making access difficult. In response, there has been an increasing number of research efforts examining the effectiveness of telehealth-delivered FBT, with a rise in studies conducted in the context of COVID-19 [32,109,111]. The results of telehealth studies have demonstrated clinically and statistically significant improvements in AN symptoms at the end of FBT [32,111,112]. Little data has demonstrated the scalability of telehealth FBT to real-world community-based health services, where most families around the world access care for their children. Further dissemination and implementation trials are required to understand the efficacy, engagement, and adoption of telehealth-delivered FBT in community-based settings. Telehealth offers a viable mechanism via which to broaden the reach, scalability and, importantly, the accessibility of FBT, which can have significant implications for the recovery trajectory of adolescents with poor access to healthcare and for generalist health systems limited in their capacity to deliver evidence-based care.

## References

[1] Jagielska G., Kacperska I. (2017). Outcome, comorbidity and prognosis in anorexia nervosa. Psychiatr. Pol..

[2] Hambleton A., Pepin G., Le A., Maloney D., Touyz S., Maguire S., National Eating Disorder Research Consortium (2022). Psychiatric and medical comorbidities of eating disorders: Findings from a rapid review of the literature. J. Eat. Disord..

[3] American Psychiatric Association (2022). Diagnostic and Statistical Manual of Mental Disorders.

[4] Golden N., Katzman D.K., Rome E., Gaete V., Nagata J., Ornstein R., Garber A., Starr T., Kohn M., Sawyer S. (2022). Medical management of restrictive eating disorders in adolescents and young adults. J. Adolesc. Health.

[5] Katzman D.K. (2005). Medical complications in adolescents with anorexia nervosa: A review of the literature. Int. J. Eat. Disord..

[6] Cost J., Krantz M.J., Mehler P.S. (2020). Medical complications of anorexia nervosa. Clevel. Clin. J. Med..

[7] Gibson D., Workman C., Mehler P.S. (2019). Medical Complications of Anorexia Nervosa and Bulimia Nervosa. Psychiatr. Clin. N. Am..

[8] Herpertz-Dahlmann B. (2008). Adolescent eating disorders: Definitions, symptomatology, epidemiology and comorbidity. Child Adolescnet Psychiatry.

[9] Molbert S.C., Klein L., Thaler A., Mohler B.J., Brozzo C., Martus P., Karnath H.O., Zipfel S., Giel K.E. (2017). Depictive and metric body size estimation in anorexia nervosa and bulimia nervosa: A systematic review and meta-analysis. Clin. Psychol. Rev..

[10] Barakat S., McLean S., Bryant E., Le A., Marks P., Aouad P., Boakes R., Brennan L., Byrne S., Caldwell B. (2023). Risk factors for eating disorders: Findings from a rapid review. J. Eat. Disord..

[11] Kuipers G., Bekker M. (2013). Attachment, mentalization and eating disorders: A review of studies using the Adult Attachment Interview. Curr. Psychiatry Rev..

[12] Goshen D., Stein D., Kurman J., Farbstein D., Enoch-Levy A., Aival-Naveh E., Gur E., Yoeli N., Bretler T., Koren D. (2023). The association between deficiencies in paternal and maternal reflective functioning and anorexia nervosa symptomatology. J. Eat. Disord..

[13] Momen N.C., Plana-Ripoll O., Yilmaz Z., Thornton L.M., McGrath J.J., Bulik C.M., Petersen L.V. (2022). Comorbidity between eating disorders and psychiatric disorders. Int. J. Eat. Disord..

[14] Steinhausen H.-C., Boyadjieva S., Griogoroiu-Serbanescu M., Neumärker K.-J. (2003). The outcome of adolescent eating disorders. Eur. Child Adolesc. Psychiatry.

[15] Kaye W., Bulik C., Thornton L., Barbarich N., Masters K. (2004). Comorbidity of Anxiety Disorders With Anorexia and Bulimia Nervosa. Am. J. Psychiatry.

[16] Bühren K., Schwarte R., Fluck F., Timmesfeld N., Krei M., Egberts K., Pfeiffer E., Fleischhaker C., Wewetzer C., Herpertz-Dahlmann B. (2014). Comorbid Psychiatric Disorders in Female Adolescents with First-Onset Anorexia Nervosa. Eur. Eat. Disord. Rev..

[17] Catone G., Patel V., Preedy V. (2021). Anorexia Nervosa and Concurrent Psychiatric Comorbidity. Eating Disorders.

[18] Preti A.R., Camboni M.V., Miorro P. (2011). A comprehensive meta-analysis of the risk of suicide in eating disorders. Acta Psychiatry Scand..

[19] Tanner A.B. (2023). Unique considerations for the medical care of restrictive eating disorders in children and young adolescents. J. Eat. Disord..

[20] Crone C., Fochtmann L., Attia E., Boland R., Escobar J., Fornari V., Golden N., Guarda A., Jackson-Triche M., Manzo L. (2023). The American Psychiatric Association Practice Guideline for the Treatment of Patients With Eating Disorders. Am. J. Psychiatry.

[21] Hay P., Chinn D., Forbes D., Madden S., Newton R., Sugenor L., Touyz S., Ward W. (2014). Royal Australian and New Zealand College of Psychiatrists: Clinical practice guidelines for the treatment of eating disorders. Aust. N. Z. J. Psychiatry.

[22] National Institute for Health and Care Excellence (2020). Eating Disorders: Recognition and Treatment. NICE Guideline [NG69].

[23] Lock J., Le Grange D. (2015). Treatment Manual for Anorexia Nervosa.

[24] Rienecke R.D., Le Grange D. (2022). The five tenets of family-based treatment for adolescent eating disorders. J. Eat. Disord..

[25] Couturier J., Kimber M., Szatmari P. (2013). Efficacy of family-based treatment for adolescents with eating disorders: A systematic review and meta-analysis. Int. J. Eat. Disord..

[26] Gorrell S., Byrne C., Trojanowski P., Fischer S., Le Grange D. (2022). A scoping review of non-specific predictors, moderators, and mediators of family-based treatment for adolescent anorexia and bulimia nervosa: A summary of the current research findings. Eat. Weight Disord.-Stud. Anorex. Bulim. Obes..

[27] Agras W., Lock J., Brandt H., Bryson S., Dodge E., Halmi K., Jo B., Johnson C., Kaye W., Wilfley D. (2014). Comparison of 2 family therapies for adolescent anorexia nervosa: A randomized parallel trial. JAMA Psychiatry.

[28] Eisler I., Simic M., Blessitt E., Dodge E. (2016). Maudsley Service Manual for Child and Adolescent Eating Disorders.

[29] Le Grange D., Crosby R., Rathouz P., Leventhal B. (2007). A randomized controlled comparison of family-based treatment and supportive psychotherapy for adolescent bulimia nervosa. Arch. Gen. Psychiatry.

[30] Le Grange D., Hughes E., Court A., Yeo M., Crosby R., Sawyer S. (2016). Randomized clinical trial of parent-focused treatment and family-based treatment for adolescent anorexia nervosa. J. Am. Acad. Child Adolesc. Psychiatry.

[31] Accurso E.C., Ciao A., Fitzsimmons-Craft E., Lock J., Le Grange D. (2014). Is weight gain really a catalyst for broader recovery?: The impact of weight gain on psychological symptoms in the treatment of adolescent anorexia nervosa. Behav. Res. Ther..

[32] Lock J., Couturier J., Matheson B., Datta N., Citron K., Sami S., Welch H., Webb C., Doxtdator K., John-Carson N. (2021). Feasibility of conducting a randomized controlled trial comparing family-based treatment via videoconferencing and online guided self-help family-based treatment for adolescent anorexia nervosa. Int. J. Eat. Disord..

[33] Lock J., Le Grange D., Agras W., Moye A., Bryson S., Jo B. (2010). Randomized clinical trial comparing family-based treatment with adolescent-focused individual therapy for adolescents with anorexia nervosa. Arch. Gen. Psychiatry.

[34] Lock J., Le Grange D., Bohon C., Matheson B., Jo B. (2023). Who Responds to an Adaptive Intervention for Adolescents With Anorexia Nervosa Being Treated With Family-Based Treatment? Outcomes From a Randomized Clinical Trial. J. Am. Acad. Child Adolesc. Psychiatry.

[35] Madden S., Miskovic-Wheatley J., Wallis A., Kohn M., Lock J., Le Grange D., Jo B., Clarke S., Rhodes P., Hay P. (2015). A randomized controlled trial of in-patient treatment for anorexia nervosa in medically unstable adolescents. Psychol. Med..

[36] Fisher C.A., Skocic S., Rutherford K., Hetrick S. (2019). Family Therapy for Those Diagnosed With Anorexia Nervosa. Cochrane Database Syst. Rev..

[37] Austin A., Anderson A., Vander Steen H., Savard C., Bergmann C., Singh M., Devoe D., Gorrell S., Patten S., Le Grange D. (2024). Efficacy of Eating Disorder Focused Family Therapy for Adolescents With Anorexia Nervosa: A Systematic Review and Meta-Analysis. Int. J. Eat. Disord..

[38] Pedersen S., Carlsson L., Bentz M. (2024). Modifications to Enhance Outcomes of Family-Based Treatment for Anorexia Nervosa: A Scoping Review. Psychiatry Int..

[39] Girz L., Lafrance Robinson A., Foroughe M., Jasper K., Boachie A. (2013). Adapting family-based therapy to a day hospital programme for adolescents with eating disorders: Preliminary outcomes and trajectories of change. J. Fam. Ther..

[40] Hurst K., Zimmer-Gembeck M. (2019). Family-based treatment with cognitive behavioural therapy for anorexia. Clin. Psychol..

[41] Peterson C., Van Diest A., Mara C., Matthews A. (2020). Dialectical behavioral therapy skills group as an adjunct to family-based therapy in adolescents with restrictive eating disorders. Eat. Disord..

[42] Lock J., Fitzpatrick K., Agras W., Weinbach N., Jo B. (2018). Feasibility Study Combining Art Therapy or Cognitive Remediation Therapy with Family-based Treatment for Adolescent Anorexia Nervosa. Eur. Eat. Disord. Rev..

[43] Lock J., Agras W., Bryson S., Kraemer H. (2005). A Comparison of Short- and Long-Term Family Therapy for Adolescent Anorexia Nervosa. J. Am. Acad. Child Adolesc. Psychiatry.

[44] Le Grange D., Huryk K., Murray S., Hughes E., Sawyer S., Loeb K. (2019). Variability in remission in family therapy for anorexia nervosa. Int. J. Eat. Disord..

[45] Couturier J., Lock J. (2006). What is recovery in adolescent anorexia nervosa?. Int. J. Eat. Disord..

[46] Lock J., Le Grange D. (2018). Family-based treatment: Where are we and where should we be going to improve recovery in child and adolescent eating disorders. Int. J. Eat. Disord..

[47] Chew C., Kelly S., Tay E., Baeg A., Khaider K., Oh J., Rajasegaran K., Saffari S., Davis C. (2021). Implementation of family-based treatment for Asian adolescents with anorexia nervosa: A consecutive cohort examination of outcomes. Int. J. Eat. Disord..

[48] Eisler I., Dare C., Russell G., Szmukler G., Le Grange D., Dodge E. (1997). Family and individual therapy in anorexia nervosa: A 5-year follow-up. Arch. Gen. Psychiatry.

[49] Eisler I., Simic M., Russell G., Dare C. (2007). A randomised controlled treatment trial of two forms of family therapy in adolescent anorexia nervosa: A five-year follow-up. J. Child Psychol. Psychiatry.

[50] Le Grange D., Accurso E.C., Lock J., Agras S., Bryson S.W. (2014). Early weight gain predicts outcome in two treatments for adolescent anorexia nervosa. Int. J. Eat. Disord..

[51] Paulson-Karlsson G., Engstrom I., Nevonen L. (2009). A pilot study of a family-based treatment for adolescent anorexia nervosa: 18-and 36-month follow-ups. Eat. Disord. J. Treat. Prev..

[52] Madden S., Miskovic-Wheatley J., Wallis A., Kohn M., Hay P., Touyz S. (2015). Early weight gain in family based treatment predicts weight gain and remission at 12-month follow-up for adolescent anorexia nervosa. Int. J. Eat. Disord..

[53] 53. Wampold B.E., Budge S.L., Laska K.M., Del Re A.C., Baardseth T.P., Flűckiger C., Minami T., Kivlighan II D.M., Gunn W. (2011). Evidence-based treatments for depression and anxiety versus treatment-as-usual: A meta-analysis of direct comparisons. Clin. Psychol. Rev..

[54] Weisz J.R., Ng M.Y., Bearman S.K. (2014). Odd Couple? Reenvisioning the Relation Between Science and Practice in the Dissemination-Implementation Era. Clin. Psychol. Sci..

[55] Lock J. (2024). Improving Access to Evidence-Based Treatments for Eating Disorders Among Youths: Where We are as a Field. Focus: J. Am. Psychiatr. Assoc..

[56] Kazdin A., Fitzimmons-Craft E., Wilfley D. (2017). Addressing critical gaps in the treatment of eating disorders. Int. J. Eat. Disord..

[57] Wilksch S. (2023). Toward a more comprehensive understanding and support of parents with a child experiencing an eating disorder. Int. J. Eat. Disord..

[58] Stewart M., Baumann O. (2024). The Effectiveness of Adolescent-Focused Therapy and Family-Based Therapy for Anorexia Nervosa. Psychol. Rep..

[59] Accurso E.C., Fitzsimmons-Craft E., Ciao A., Le Grange D. (2015). From efficacy to effectiveness: Comparing outcomes for youth with anorexia nervosa treated in research trials versus clinical care. Behav. Res. Ther..

[60] Le Grange D., Gorrell S., Hughes E., Accurso E., Yeo M., Pradel M., Sawyer S. (2020). Delivery of Family-Based Treatment for Adolescent Anorexia Nervosa in a Public Health Care Setting: Research Versus Non-Research Specialty Care. Front. Psychiatry.

[61] Couturier J., Kimber M. (2015). Dissemination and Implementation of Manualized Family-Based Treatment: A Systematic Review. Eat. Disord..

[62] Simic M., Stewart C.S., Konstantellou A., Hodsoll J., Eisler I., Baudinet J. (2022). From efficacy to effectiveness: Child and adolescent eating disorder treatments in the real world (part 1)—Treatment course and outcomes. J. Eat. Disord..

[63] Hughes E., Le Grange D., Court A., Yeo M., Campbell S., Whitelaw M., Atkins L., Sawyer S. (2014). Implementation of Family-Based Treatment for Adolescents With Anorexia Nervosa. J. Pediatr. Health Care.

[64] Von Ranson K., Wallace L., Stevenson A. (2013). Psychotherapies provided for eating disorders by community clinicians: Infrequent use of evidence-based treatment. Psychother. Res..

[65] Hughes E.K., Poker S., Bortz A., Yeo M., Telfer M., Sawyer S.M. (2020). Adolescent and Parent Experience of Care at a Family-Based Treatment Service for Eating Disorders. Front. Psychiatry.

[66] Conti J., Joyce C., Natoli S., Skeoch K., Hay P. (2021). “I’m still here, but no one hears you”: A qualitative study of young women’s experiences of persistent distress post family-based treatment for adolescent anorexia nervosa. J. Eat. Disord..

[67] Wufong E., Rhodes P., Conti J. (2019). "We don’t really know what else we can do": Parent experiences when adolescent distress persists after the Maudsley and family-based therapies for anorexia nervosa. J. Eat. Disord..

[68] Couturier J., Kimber M., Jack S., Niccols A., Van Blyderveen S., McVey G. (2013). Understanding the uptake of family-based treatment for adolescents with anorexia nervosa: Therapist perspectives. Int. J. Eat. Disord..

[69] Couturier J., Lock J., Kimber M., McVey G., Barwick M., Niccols A., Webb C., Findlay S., Woodford T. (2017). Themes arising in clinical consultation for therapists implementing family-based treatment for adolescents with anorexia nervosa: A qualitative study. J. Eat. Disord..

[70] Egbert A., Irizarry B., Lualdi E., Tortolani C., Donaldson D., Goldschmidt A. (2024). A qualitative assessment of provider-perceived barriers to implementing family-based treatment for anorexia nervosa in low-income community settings. J. Eat. Disord..

[71] Dimitropoulos G., Lock J., Agras W., Brandt H., Halmi K., Jo B., Kaye W., Pinhas L., Wilfley D., Woodside B. (2020). Therapist adherence to family-based treatment for adolescents with anorexia nervosa: A multi-site exploratory study. Eur. Eat. Disord. Rev..

[72] Fonagy P., Luyten P. (2019). Fidelity vs. flexibility in the implementation of psychotherapies: Time to move on. World Psychiatry.

[73] Kosmerly S., Waller G., Robinson A.L. (2015). Clinician adherence to guidelines in the delivery of family-based therapy for eating disorders. Int. J. Eat. Disord..

[74] Aradas J., Sales D., Rhodes P., Conti J. (2019). “As long as they eat”? Therapist experiences, dilemmas and identity negotiations of Maudsley and family-based therapy for anorexia nervosa. J. Eat. Disord..

[75] Accurso E.C., Le Grange D., Graham A. (2020). Attitudes Toward Family-Based Treatment Impact Therapists’ Intent to Change Their Therapeutic Practice for Adolescent Anorexia Nervosa. Front. Psychiatry.

[76] Couturier J., Kimber M., Barwick M., McVey G., Findlay S., Webb C., Niccols A., Lock J. (2021). Assessing fidelity to family-based treatment: An exploratory examination of expert, therapist, parent, and peer ratings. J. Eat. Disord..

[77] Waller G., Turner H. (2016). Therapist drift redux: Why well-meaning clinicians fail to deliver evidence-based therapy, and how to get back on track. Behav. Res. Ther..

[78] Fox J.R., Dean M., Whittlesea A. (2017). The experience of caring for or living with an individual with an eating disorder: A meta-synthesis of qualitative studies: Experience of caring for someone with AN. Clin. Psychol. Psychother..

[79] Thibault I., Leduc K., Tougas A., Pesant C. (2023). “For me, it’s a real pain”: A Foray into the Experience of Parents Involved in Family-Based Treatment. Fam. J..

[80] Wallis A., Miskovic-Wheatley J., Madden S., Rhodes P., Crosby R., Cao L., Touyz S. (2017). How does family functioning effect the outcome of family based treatment for adolescents with severe anorexia nervosa?. J. Eat. Disord..

[81] 81. Le Grange D., Lock J., Agras W.S., Moye A., Bryson S.W., Jo B., Kraemer H.C. (2012). Moderators and mediators of remission in family-based treatment and adolescent focused therapy for anorexia nervosa. Behav. Res. Ther..

[82] Lock J., Couturier J., Bryson S., Agras S. (2006). Predictors of dropout and remission in family therapy for adolescent anorexia nervosa in a randomized clinical trial. Int. J. Eat. Disord..

[83] Borges R., Crest P., Landsverk J., Accurso E.C. (2024). Adaptations to family-based treatment for Medicaid-insured adolescents with anorexia nervosa. Front. Psychol..

[84] Dimitropoulos G., Singh M., Sauerwein J., Pedram P., Kimber M., Pradel M., Eckhardt S., Forsberg S., Keery H., Allan E. (2024). Examining clinicians’ perceptions and experiences working with diverse families in family-based treatment: Common adaptations and considerations for treatment engagement. Int. J. Eat. Disord..

[85] Japanese Society of Eating Disorders (2012). Sesshoku Shougai Chiryou Gaidorain. Treat. Guidel. Eat. Disord..

[86] Iguchi T., Miyawaki D., Harada T., Ogiwara K. (2021). Introduction of family-based treatment to Japan with adaptations to optimize the cultural acceptability and advance current traditional treatments of adolescent anorexia nervosa. Int. J. Eat. Disord..

[87] Accurso E.C., Buckelew S., Snowden L. (2021). Youth insured by medicaid with restrictive eating disorders—Underrecognized and underresourced. JAMA Pediatr..

[88] Deloitte Access Economics (The Butterfly Foundation) (2024). Paying the Price, Second Edition: The Economic and Social Impact of Eating Disorders in Australia.

[89] Mitchison D., Hay P., Slewa-Younan S., Mond J. (2014). The changing demographic profile of eating disorder behaviors in the community. BMC Public Health.

[90] Australian Institute of Health and Welfare (2018). Rural, Regional And Remote Health: Indicators of Health Status and Determinants of Health.

[91] Hahn S.L., Burnette C.B., Borton K.A., Mitchell Carpenter L., Sonneville K.R., Bailey B. (2023). Eating disorder risk in rural US adolescents: What do we know and where do we go?. Int. J. Eat. Disord..

[92] Alman J., Hoiles K., Watson H., Egan S., Hamilton M., McCormack J., Potts J., Forbes D., Shu C. (2014). A decade of data from a specialist statewide child and adolescent eating disorder service: Does local service access correspond with the severity of medical and eating disorder symptoms at presentation?. J. Eat. Disord..

[93] Johnson C., Cook L., Cadman K., Andersen T., Williamson P., Wade T. (2022). Evaluating an implementation model of evidence-based therapy for eating disorders in non-specialist regional mental health settings. J. Eat. Disord..

[94] McCormack J., Dale N., Watson H., Forster M., Alderson K. Building community capacity and improving efficiencies for rural and remote patients with eating disorders: Innovative treatment pathways. Proceedings of the 6th Annual Conference of the Australia and New Zealand Academy for Eating Disorders.

[95] Sheridan T., Brown L., Moy S., Harris D. (2013). Health outcomes of eating disorder clients in a rural setting. Aust. J. Rural Health.

[96] Weber M., Davis K. (2012). Food for thought: Enabling and constraining factors for effective rural eating disorder service delivery. Aust. J. Rural Health.

[97] Sonneville K.R., Lipson S.K. (2018). Disparities in eating disorder diagnosis and treatment according to weight status, race/ethnicity, socioeconomic background, and sex among college students. Int. J. Eat. Disord..

[98] Wade T., Johnson C., Cadman K., Cook L. (2022). Turning eating disorders screening in primary practice into treatment: A clinical practice approach. Int. J. Eat. Disord..

[99] Butterfly Foundation (The Butterfly Foundation) (2020). Maydays 2020 Survey Report: Barriers to Accessing Eating Disorder Healthcare And Support.

[100] McCormack J., Watson H., Harris C., Potts J., Forbes D. (2013). A hub and spokes approach to building community capacityfor eating disorders in rural Western Australia. Aust. J. Rural Health.

[101] Moffatt J., Eley D. (2010). The reported benefits of telehealth for rural Australians. Aust. Health Rev..

[102] Aardoom J.J., Dingemans A.E., Spinhoven P., Van Furth E. (2013). Treating eating disorders over the internet: A systematic review and future research directions. Int. J. Eat. Disord..

[103] Rutledge C.M., Kott K., Schweickert P.A., Poston R., Fowler C., Haney T.S. (2017). Telehealth and eHealth in nurse practitioner training: Current perspectives. Adv. Med. Educ. Pract.

[104] Fisk M., Livingstone A., Pit S. (2020). Telehealth in the Context of COVID-19:Changing Perspectivesin Australia, the United Kingdom, and the United States. J. Med. Internet Res..

[105] Keuroghlian A.S., Marcus P.H., Neufeld J., Phillips E., Grasso C., Wozniak J.R. (2023). Telehealth for psychiatry and mental healthcare can improve access and patient outcomes. Nat. Med..

[106] Sproch L., Anderson K. (2019). Clinician-delivered Teletherapy for eating disorders. Psychiatr. Clin..

[107] Mitchell J., Crosby R., Wonderlich S., Crow S., Lancaster K., Simonich H., Swan-Kremeier L., Lysne C., Myers T. (2008). A randomized trial comparing the efficacy ofcognitive–behavioral therapy for bulimia nervosa delivered viatelemedicine versus face-to-face. Behav. Res. Ther..

[108] Gorrell S., Reilly E., Brosof L., Le Grange D. (2022). Use of Telehealth in the Management of Adolescent Eating Disorders: Patient Perspectives and Future Directions Suggested from the COVID-19 Pandemic. Adolesc. Health Med. Ther..

[109] Anderson K., Byrne C., Crosby R., Le Grange D. (2017). Utilizing telehealth to deliver family-based treatment for adolescent anorexia nervosa. Int. J. Eat. Disord..

[110] Goldfield G., Boachie A. (2003). Delivery of family therapy in thetreatment of anorexia nervosa using Telehealth. Telemed. J. E-Health.

[111] Couturier J., Pellegrini D., Grennan L., Nicula M., Miller C., Agar P., Webb C., Anderson K., Barwick M., Dimitropoulos G. (2023). Multidisciplinary implementation of family-based treatment delivered by videoconferencing (FBT-V) for adolescent anorexia nervosa during the COVID-19 pandemic. Transl. Behav. Med..

[112] Hambleton A., Le Grange D., Kim M., Miskovic-Wheatley J., Touyz S., Maguire S. (2024). Delivering evidence-based treatment via telehealth for Anorexia Nervosa in rural health settings: A multi-site feasibility implementation study. J. Eat. Disord..

[113] Van Huysse J.L., Prohaska N., Miller C., Jary J., Sturza J., Etsell K., Bravender T. (2023). Adolescent eating disorder treatment outcomes of an in-person partial hospital program versus a virtual intensive outpatient program. Int. J. Eat. Disord..

[114] Stewart C.S., Konstantellou A., Kassamali F., McLaughlin N., Cutinha D., Bryant-Waugh R., Simic M., Eisler I., Baudinet J. (2021). Is this the ‘new normal’? A mixed method investigation of young person, parent and clinician experience of online eating disorder treatment during the COVID-19 pandemic. J. Eat. Disord..

[115] Couturier J., Pellegrini D., Grennan L., Nicula M., Miller C., Agar P., Webb C., Anderson K., Barwick M., Dimitropoulos G. (2022). A qualitative evaluation of team and family perceptions of family-based treatment delivered by videoconferencing (FBT-V) for adolescent Anorexia Nervosa during the COVID-19 pandemic. J. Eat. Disord..

[116] Baudinet J., Konstantellou A., Hambleton A., Bialluch K., Hurford G., Stewart C.S. (2023). Do People Want the ‘New Normal’? A Mixed Method Investigation of Young Person, Parent, and Clinician Experience and Preferences for Eating Disorder Treatment Delivery in the Post-COVID-19 World. Nutrients.

[117] Culbert K.M., Racine S.E., Klump K.L. (2015). Research Review: What we have learned about the causes of eating disorders-a synthesis of sociocultural, psychological, and biological research. J. Child Psychol. Psychiatry.

[118] Del Casale A., Adriani B., Modesti M.N., Virzì S., Parmigiani G., Vento A.E., Speranza A.M. (2023). Anorexia nervosa and familial risk factors: A systematic review of the literature. Curr. Psychol..

[119] Astrachan-Fletcher E., Accurso E.C., Rossman S., McClanahan S.F., Dimitropoulos G., Le Grange D. (2018). An exploratory study of challenges and successes in implementing adapted family-based treatment in a community setting. J. Eat. Disord..

